# Characterization of the Human Papillomavirus 16 Oncogenes in K14HPV16 Mice: Sublineage A1 Drives Multi-Organ Carcinogenesis

**DOI:** 10.3390/ijms232012371

**Published:** 2022-10-15

**Authors:** Daniela Cochicho, Alexandra Nunes, João Paulo Gomes, Luís Martins, Mário Cunha, Beatriz Medeiros-Fonseca, Paula Oliveira, Margarida M. S. M. Bastos, Rui Medeiros, Joana Mendonça, Luis Vieira, Rui M. Gil da Costa, Ana Felix

**Affiliations:** 1 NOVA Medical School-Universidade NOVA de Lisboa, NOVA University, 1169-056 Lisbon, Portugal; 2 Virology Laboratory from Clinical Pathology Department, Portuguese Institute of Oncology Francisco Gentil (IPOLFG), 1099-023 Lisbon, Portugal; 3 Genomics and Bioinformatics Unit, Department of Infectious Diseases, National Institute of Health Dr. Ricardo Jorge (INSA), 1649-016 Lisbon, Portugal; 4 Faculty of Veterinary Medicine, Lusófona University, Campo Grande 376, 1749-024 Lisbon, Portugal; 5 Centre for the Research and Technology of Agro-Environmental and Biological Sciences (CITAB), Inov4Agro, University of Trás-os-Montes e Alto Douro (UTAD), Quinta de Prados, 5000-801 Vila Real, Portugal; 6 Laboratory for Process Engineering, Environment, Biotechnology and Energy (LEPABE), Faculty of Engineering, University of Porto, Rua Dr. Roberto Frias, 4200-465 Porto, Portugal; 7 Associate Laboratory in Chemical Engineering, Faculty of Engineering (ALiCE), University of Porto, Rua Dr. Roberto Frias, 4200-465 Porto, Portugal; 8 Molecular Oncology and Viral Pathology Group, Portuguese Oncology Institute of Porto (IPO Porto), Rua Dr. António Bernardino de Almeida, 4200-072 Porto, Portugal; 9 Health Research Network, Research Center of Portuguese Oncology Institute of Porto (CI-IPOP/RISE@CI-IPOP), Rua Dr. António Bernardino de Almeida, 4200-072 Porto, Portugal; 10 Technology and Innovation Unit, Department of Human Genetics, National Institute of Health, Av. Padre Cruz (INSA), 1649-016 Lisbon, Portugal; 11 Post-Graduate Programme in Adult Health (PPGSAD), Morphology Department, University Hospital (HUUFMA), Federal University of Maranhão, São Luís 65085-580, Brazil; 12 Pathology Department, Portuguese Institute of Oncology Francisco Gentil, 1099-023 Lisbon, Portugal

**Keywords:** K14HPV16, carcinogenesis, HPV16, variant, lineage

## Abstract

The study of human papillomavirus (HPV)-induced carcinogenesis uses multiple in vivo mouse models, one of which relies on the cytokeratin 14 gene promoter to drive the expression of all HPV early oncogenes. This study aimed to determine the HPV16 variant and sublineage present in the K14HPV16 mouse model. This information can be considered of great importance to further enhance this K14HPV16 model as an essential research tool and optimize its use for basic and translational studies. Our study evaluated HPV DNA from 17 samples isolated from 4 animals, both wild-type (n = 2) and HPV16-transgenic mice (n = 2). Total DNA was extracted from tissues and the detection of HPV16 was performed using a qPCR multiplex. HPV16-positive samples were subsequently whole-genome sequenced by next-generation sequencing techniques. The phylogenetic positioning clearly shows K14HPV16 samples clustering together in the sub-lineage A1 (NC001526.4). A comparative genome analysis of K14HPV16 samples revealed three mutations to the human papillomaviruses type 16 sublineage A1 representative strain. Knowledge of the HPV 16 variant is fundamental, and these findings will allow the rational use of this animal model to explore the role of the A1 sublineage in HPV-driven cancer.

## 1. Introduction

**Figure 1:**
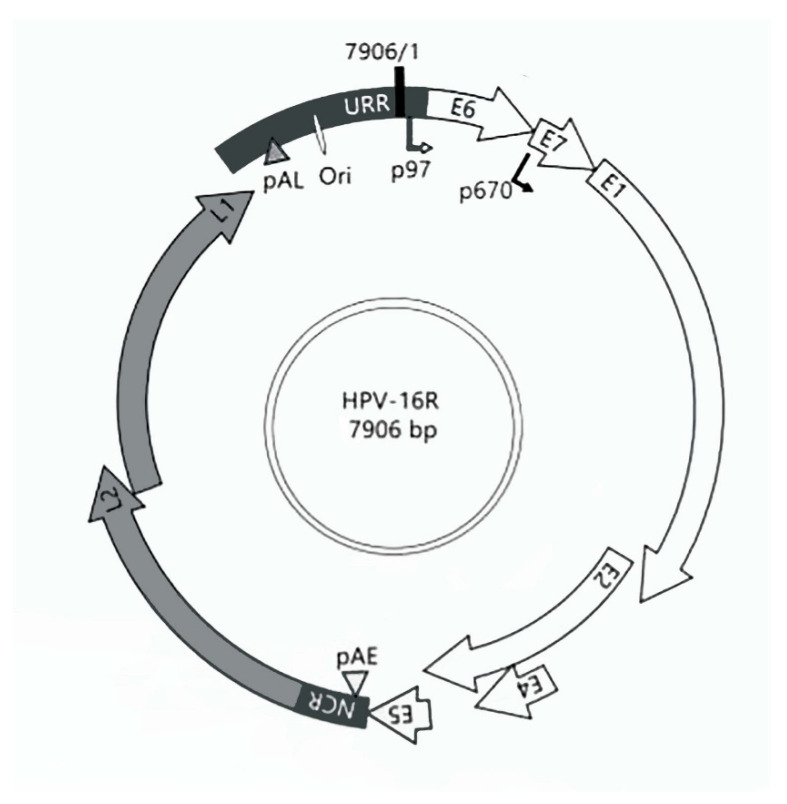
Schematic of the HPV-16R genome. The genome organization of 7906 base pairs (bp) arranged in a circular form represents the three functional regions, early, late, and long control (LCR); and a non-coding region (NCR). These are separated by two polyadenylation sites, described as early (pAE) and late (pAL). In total, the genome encodes eight open reading frames (ORFs), of which E1, E2, E4, E5, E6, and E7 are from the early region; and L1 and L2 are from the late region. The LCR, located between the E6 and L1 gene, is a regulatory region that includes the origin of replication (ori) and the p97 promoter.

Papillomaviruses are species-specific double-stranded DNA viruses of 8000 base pairs (bp) length (Figure 1) that have preferential tropism for epithelial cells. Infection with human papillomavirus (HPV) is the most common sexually transmissible infection and induces a range of benign (e.g., condylomas) and malignant lesions, such as cervical cancer and other anogenital squamous cell carcinomas and a growing subset of oropharyngeal squamous cell carcinomas [1]. HPV can be classified into genotypes defined by a greater than 10.0% variation in their L1 gene sequence [2]. Currently, over 200 HPV types are recognized and grouped as high-risk (e.g., HPV16 and HPV18, associated with malignant cervical lesions) or low-risk (HPV6 and HPV11, associated with benign lesions) [2,3,4]. Accumulating data indicate that HPV intra-type variants, lineages (defined by a 1.0–10.0% genomic variation), and sub-lineages (0.5–1.0% variation) may differ in their carcinogenic potential [5,6,7,8], as recently reviewed [9,10]. The study of HPV-induced carcinogenesis uses multiple in vivo mouse models [11], one of which relies on the Krt14 (cytokeratin 14) gene promoter to drive the expression of all HPV16 early oncogenes and specifically target basal keratinocytes (known as K14HPV16 mice) [12]. This widely used model was first developed in the 1990s and has since been used to model cervical cancer [13] and other HPV16-associated malignancies [14,15,16]. This animal model has also proved useful to elucidate the immune-modulatory mechanisms involved in HPV16-induced cancers and to test potential new therapies. However, the specific variant and sub-lineage involved in K14HPV16 mice remain unknown [12]. Determining which variant and sublineage are present in this animal model would help test their potential associations with the development of cancer at specific locations. Additionally, this information would help researchers. This study aimed to determine the HPV16 variant and sublineage present in the K14HPV16 mouse model, further characterizing this important research tool and potentiating its use for basic and translational studies.

## 2. Results

### 2.1. Sample Characterization

**Figure 2:**
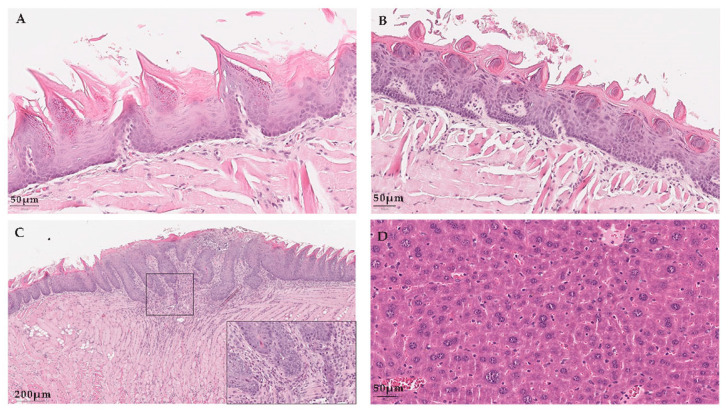
Histological findings of WT and MUT tongue and oral cancer. Legend: Histological samples from wild-type and transgenic K14HPV16 mice stained with H&E. (**A**) low power view of normal squamous cell epithelium of the tongue from a wild-type mouse; (**B**) low power view of mild dysplastic squamous cell epithelium with enlarged hyperchromatic nuclei in the upper layers of the epithelium from a transgenic K14HPV16 mouse and (**C**) low power view of a focally invasive squamous cell carcinoma with thickened rete ridges and dysplastic cells with hyperchromatic nuclei, irregular basement membrane and focally disrupted associated with inflammatory cells in the stroma from a transgenic K14HPV16 mouse; the inset shows the invasive front. (**D**) low power view of the liver of one transgenic K14HPV16 mouse showing normal structure and cytology.

Histological samples from wild-type (WT) animals showed normal histology and HPV16-transgenic (MUT) mice samples showed typical proliferative epithelial lesions of the skin and tongue, while the liver and lymph node samples from WT and HPV16-trangenic mice showed mild inflammatory changes or none at all. The oral tumor found in the HPV16-trangenic mice was identified as a squamous cell carcinoma, with minimal invasion of the tongue (Figure 2).

### 2.2. HPV16 Genome Coverage and Quality

Among the nine HPV16-positive samples, the mean depth of coverage was 2080-fold, ranging between 870-fold for the node sample and 3726-fold for the tumor sample. On average, samples had 75% of the HPV16 genome covered, with 95% of it covered by at least 10-fold. The only exception was a ~1900 bp region comprising most of the L1 gene, the upstream regulatory region (URR) and the beginning of the E6 gene for which no amplification signal was observed for all samples. This is in agreement with the model design [12] which encompasses the entire HPV16 early coding region from bp 97 to 6152, although the gap between the L1 and E6 region (~1945 pb) compromises the hybridization efficiency of specific primers within the primer pool for that region.

### 2.3. K14HPV16 Lineage Classification

**Figure 3:**
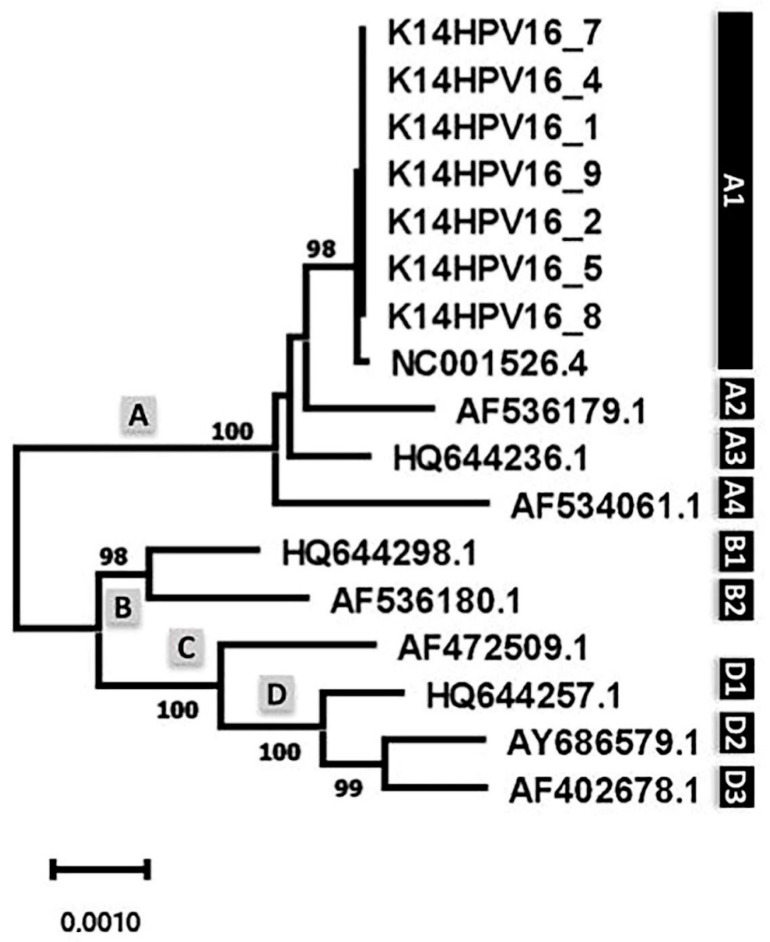
Phylogenetic positioning of K14HPV16 samples within the major HPV16 lineages (A–D). Legend: the phylogenetic tree was generated using the neighbor-joining method [18] with the maximum composite likelihood model [17] and depicts the genetic relationships of the obtained K14HPV16 genome consensus sequences to each representative genome. Numbers next to the branch nodes indicate the bootstrap values (1000 replicates). HPV16 lineages are shown in grey boxes next to the main tree branches, while sublineages (A1–A4, B1, B2, and D1–D3) are illustrated in black boxes.

HPV16 variants have been classified into four major lineages (A–D) and sublineages (A1–A4, B1, B2, and D1–D3), based on their genome sequence diversity [17]. To determine the phylogenetic positioning of the HPV16 transgenes in K14HPV16 samples, the respective consensus sequences were aligned against the genomes of lineage/sublineage representative sequences (Supplementary Table S1). For K14HPV16-3 and K14HPV16-6, the generation of a consensus sequence failed (% of genome covered <70%), and those two samples were not included in the phylogenetic analysis. Nevertheless, they share the same major mutations as the remaining K14HPV16 samples against the HPV16 sublineage A1 representative strain (see details below). The phylogenetic analysis (Figure 3) clearly shows K14HPV16 samples clustering together in the sub-lineage A1 (NC001526.4).

### 2.4. HPV16 Sequencing

Comparative genome analysis of K14HPV16 samples revealed a total of three mutations to the HPV16 sublineage A1 representative strain. All mutations fall within the coding region of the E1 early gene, with more than half yielding amino acid changes. Two of these (330A > G|Leu110Leu and 978A > G|Ile326Met) have become evolutionarily fixed (frequency of 100%) based on the observed deep coverage supporting each alteration. Indeed, 100% of the reads mapping each position (for pos 330: ranging from 1580x for sample 6 to 15456x for sample 8; and for pos. 978; ranging from 636x for sample 6 to 11951x for sample 8) support the two variants found in all K14HPV16 samples. The last mutation is a non-synonymous minor intra-patient single nucleotide variant (166A > T|Asn56Tyr) exhibiting differential intermediate frequencies across the nine K14HPV16 samples (from 12.3% for K14HPV16_15 to 22.1% for K14HPV16_11).

## 3. Discussion

HPV16 variants show a differential prevalence in diverse geographical locations and are associated with differential oncogenic potential [5,6,7,8]. Caucasian women infected with the A1/A2 variant are at a higher risk of CIN3+ compared to women of other genetic backgrounds [19]. This is also the most common variant found in head and neck oral cancers in Caucasians and cervical carcinomas [20], suggesting a heightened oncogenic potential compared with other HPV16 variants. 

For the first time, the present study determined the variant lineage present in the K14HPV16 transgenic mouse model, based on the relatedness of reference sequences including ten HPV16 A, B, C, and D variant lineages. The K14HPV16 mouse model was shown to carry the HPV16 A1 sublineage. Despite their isolation from distinct tissues, the K14HPV16 samples were found to be highly genetically related among them, only differing by a mean of 1.0 ± 0.8 nucleotides and showing a mean distance of 3.1 ± 1.7 nucleotides from the phylogenetically closest sublineage A1 representative strain. On the other hand, K14HPV16 samples exhibited a mean distance to other sublineages that ranged from 22.0 ± 4.5 (sublineage A3) to 112.0 ± 10.3 (sublineage D2). Considering that variant HPV sublineages are empirically defined as exhibiting genome differences in the 0.5–1.0% range [21], respectively, these results point to a clonal origin of K14HPV16 samples, in agreement with the congenic nature of this mouse strain [12]. 

Having determined the variant lineage and sublineage present in K14HPV16 mice, it is now possible to discuss its association with the various kinds of neoplastic lesions observed in this mouse strain. K14HPV16 mice have been used to mimic the development of cervical cancer in the 1990s [13]. In this study, the HPV16 oncogenes were necessary but insufficient to induce cervical cancer and chronic estrogen supplementation was required for carcinogenesis. Based on the present results, it is interesting to speculate that, although the A1 sublineage is clinically associated with a heightened risk of cervical cancer, it may require hormonal co-factors for efficient carcinogenesis. Recently, our group employed K14HPV16 mice for producing the first mouse model of HPV-related penile cancer [22]. Again, the A1 lineage was necessary but insufficient to induce invasive squamous cell carcinoma and a tobacco-related co-carcinogen was needed. However, the HPV16 A1 lineage was able to induce a range of penile intraepithelial lesions in this model without additional co-factors. Interestingly, K14HPV16 mice develop squamous cell carcinomas specifically located at the tongue base, without the need for any chemical or hormonal co-carcinogens—although tumors were more frequent in female mice [15]. Oropharyngeal cancer incidence could be increased in this model by exposure to ptaquiloside, a bracken fern carcinogen, suggesting a synergistic effect [23]. Based on these observations, we speculate that the oncogenic potential of the HPV16 A1 variant lineage in this mouse model is dependent on the anatomic site: cervical and penile carcinogenesis require the presence of co-factors while oropharyngeal cancer may be solely induced by the viral oncogenes, although the predominant incidence in female mice suggests a role of the higher physiological state of estrogen. Further studies are warranted to examine the interplay between different HPV16 lineages and hormonal co-factors. Remarkably, another study using mice carrying only the HPV16 E6 and E7 transgenes without variant assignment found that oropharyngeal carcinogenesis depended on a chemical co-carcinogen [24]. 

The K14HPV16 mouse model was also used to test new therapies and cancer preventive strategies by our group and others [14,16,25,26]. In general, the development of lesions in this animal model is strongly associated with modulation of the immune response within the tumor microenvironment, and combined immune therapy approaches are required to prevent lesion development [26]. Now, it will be possible to assess the specific impact of those approaches on the HPV16 A1 sublineage, and it would be desirable to develop animal models that represented other HPV16 strains as well. 

From the comparative analysis of the genome, a total of three variants in the early E1 region were validated, two of which had a missense effect. However, little can be concluded about the clinical significance of these variants in our series. In fact, as previously reported for the K14HPV16 model, the function of E1 and E2 gene products does not influence in terms of severity or extent of progression of the hyperplastic/dysplastic phenotype [12] described in a spectrum of pathologies such the skin, mucosa, and anal squamous epithelium.

The knowledge of HPV variants is very important as it is recognized that minor changes in the HPV sequence affect its carcinogenic potential [18]. Our study identified that K14HPV16 FVB/n mice harbor the HPV16 variant sublineage A1, the most common variant associated with cervical and oropharyngeal neoplasia in cancer patients. This result will allow the rational use of this animal model to explore the role of the A1 sublineage in HPV-driven cancer. Experimental results from the K14HPV16 mouse model suggest that the HPV16 A1 sublineage is associated with a high incidence of tongue base cancer in this model, while the development of cervical and penile cancers requires the action of chemical co-factors, leading us to speculate that different organ sites/microenvironments can modulate the oncogenic potential of this sublineage. However, additional studies are required to test this hypothesis.

## 4. Material and Methods

### 4.1. Animals

HPV16-transgenic mice in the FVB/n strain background—FVB.Cg-Tg(KRT14-HPV16)wt1Dh strain known as K14HPV16 mice—were generously donated by Drs. Jeffrey Arbeit and Douglas Hanahan of the University of California through the Mouse Repository of the US National Cancer Institute. Generation of K14HPV16 mice has been previously reported [12]. These animals express all early HPV16 genes under the control of the cytokeratin 14 (KRT14) gene promoter and develop cervical and cutaneous lesions resembling those of cancer patients [12,13]. The experiments were approved by the University of Trás-os-Montes and Alto Douro Ethics Committee (Approval Number 10/2013) and the Portuguese Veterinary Directorate (Approval Number 0421000/000/2014). Mice were maintained and bred according to the Portuguese (Decree-Law 1005/92 dated October the 23rd) and European (EU Directive 2010/63/EU) legislation, with 12 h light/12 h dark cycles, at 20–24 °C, and 50 ± 10% relative humidity. At 30 weeks old, 2 HPV16-transgenic and 2 matched wild-type female mice were sacrificed by intraperitoneal administration of a mixture of xylazine and ketamine, followed by cardiac puncture exsanguination, according to FELASA guidelines [27].

### 4.2. Samples

A total of 17 samples from 4 animals were studied, both wild-type (WT, *n* = 2) and HPV16-transgenic mice (MUT, *n* = 2). The distribution of samples by the animal condition is presented in Supplementary material Table S1. Overall, the samples collected from tongue (*n* = 4), skin (*n* = 4), liver (*n* = 4), lymph node (*n* = 4), and oral tumor (*n* = 1) were evaluated/analyzed. The samples were snap-frozen in liquid nitrogen and stored at −80 °C. Matched samples were fixed in formalin and processed for histological evaluation.

### 4.3. Histological Processing

The samples for histological analysis were fixed in 10% neutral buffered formalin and paraffin-embedded. Five micrometer-thick sections were stained with hematoxylin and eosin for histological classification, as previously described [13,14,21,28].

### 4.4. HPV16 Genotyping 

Total DNA was extracted using the universal acid nucleic extraction kit (Seegene). The detection of HPV16 was performed using a qPCR multiplex (Anyplex™ II HPV28 Detection, Seegene) assay able to detect 28 HPV types. Amplification was performed in a CFX96 device (Bio-Rad). No-target controls were used for each reaction. HPV16-positive samples were subsequently submitted to whole-genome sequencing using next-generation sequencing (NGS) techniques.

### 4.5. HPV16 Whole-Genome Sequencing

The complete 8 kb genomes were amplified by PCR, under 4 pools of primers developed as previously described [29], using a Q5 Hot Start High-Fidelity 2X Master Mix DNA Polymerase (New England Biolabs). PCR products were evaluated for size and specificity by agarose gel analysis. The four independent PCR products of each sample were pooled together in approximately equal molar amounts based on the intensity of bands after electrophoresis. The pooled amplicons were purified from small fragments and primers using the AMPure XP beads (Beckman Coulter) according to the manufacturer’s instructions before library preparation. The Nextera XT DNA library prep kit (Illumina) was used to prepare bead-based normalized dual-indexed libraries for sequencing. The library pool was sequenced on a MiSeq benchtop sequencer (Illumina) using a reagent kit V3. A sequencing strategy with 250-bp paired-end reads was used. The INSaFLU (https://insaflu.insa.pt/) (accessed on 15 July 2022), an online platform for amplicon-based next-generation sequencing data analysis [30], was used for the reads’ quality control, variant detection/inspection, and sequence consensus generation. The genome sequence of a representative of HPV16 sublineage A1 (GenBank accession number NC001526.4) was used as a reference for mapping and single-nucleotide variant (SNV) annotation. Regions with a depth of coverage below 10-fold were automatically masked in the INSaFLU pipeline by placing undefined bases “N” in the consensus sequence. Low-coverage regions were visually inspected using Integrative Genomics Viewer (IGV). SNVs were assumed in consensus when they displayed more than 50% intra-sample frequency. MEGA11 software (http://www.megasoftware.net) (accessed on 4 September 2022) [17] was applied to calculate the matrices of nucleotide distances and to perform phylogenetic reconstructions. Genome sequences of representative strains from other HPV16 lineages and sublineages (Supplementary material Table S2) were also included in the analyses.

### 4.6. Variation Sites and their Association with Previous Studies

The SNV data were cross-referenced using the online dbSNP-PUBMED database (https://www.ncbi.nlm.nih.gov/snp/; accessed 28 January 2022) and also compared with previous HPV16 variant published data [31,32,33,34,35,36,37,38,39,40,41,42,43,44,45,46,47,48,49,50,51] that describe variation sites within whole-genome coding regions (Supplementary Table S3). Included articles were selected from a previously described systematic review [9] and all data from the articles that describe the mutations identified in the head and neck were analyzed; additionally, those identified in the pathology of the cervix were also included.

## Data Availability

The authors confirm that the data supporting the findings of this study are available within the article and its Supplementary materials.

## Supplementary Materials

The following supporting information can be downloaded at: https://www.mdpi.com/article/10.3390/ijms232012371/s1.
