# Supplementary material for: Characterization of the Human Papillomavirus 16 Oncogenes in K14HPV16 Mice: Sublineage A1 Drives Multi-Organ Carcinogenesis
Source: Int J Mol Sci. 2022 Oct 15;23(20):12371. doi: 10.3390/ijms232012371 (PMC9604181; doi:10.3390/ijms232012371)
Supplement: Supplementary file 1 [file ijms-23-12371-s001.zip › Suplementary Table S3. Previous studies from HPV16 lineages and sublineages.pdf]

Table S3. Previous studies from HPV16 lineages and sublineages

REF.	population	Local	HPV region	E1	E1	E1	E1	E2	E4	E2	E2	E5	NC	L2	L2	L1
				**166 A>T**	**330 A>G**	**978 A>G**	**1076 delA**	**607 A>G**	**174 C>A**	**761 C>A**	**1023 G>T**	**159 A>G**	**3364 T>C**	**618 C>T**	**1293 T>A**	**46 G>A**
[5]	Brazil	cervix	L1 E6 LCR													
[6]	Sweden	tonsilar	E6													
[19]	UK	cervix	E2													
[28]	China	cervix	E1 E2			**x**										
[31]]	Mexico	cervix	E6													
[32]	Japan	cervix	E1 E2													
[33]	USA	cervix	E6 L1 L2													
[34]	Netherlands	cervix	WG													
[35]	USA	oropharynx	E6 E7													
[36]	China	cervix	LCR													
[37]	Mexico	cervix	L1 E6 LCR													
[38]	Mexico	cervix	E6													
[39]	China	cervix	E2 E6 E7													
[40]	Brazil	cervix	WG													
[41]	USA	cervix	E6													
[42]	Germany	HN	E6 E7													
[43]	Japan	cervix	WG													
[44]	USA	cervix	E2 E7													
[45]	USA	cervix	E2 E7													
[46]	Germany	HN	E6 E7													
[47]	Korea	cervix	E6 E7 L1 LCR													
[48]	China	cervix	E6 E7													
[49]	USA	cervix|HN	E6 E7													
[50]	India	breast|cervix	E6 E7 ORF													
[51]	Tunisia	cervix	E2													

legend: NC non coding region
