# Supplementary material for: Characterization of the Human Papillomavirus 16 Oncogenes in K14HPV16 Mice: Sublineage A1 Drives Multi-Organ Carcinogenesis
Source: Int J Mol Sci. 2022 Oct 15;23(20):12371. doi: 10.3390/ijms232012371 (PMC9604181; doi:10.3390/ijms232012371)
Supplement: Supplementary file 1 [file ijms-23-12371-s001.zip › Supplementary Table S1 Characterization of mouse samples.pdf]

Table S1. Characterization of mouse samples.

Sample	Animal	Sample tissue	HPV DNA
1	I WT	skin	not detected
2		liver	not detected
3		lymph node	not detected
4		tongue	not detected
5	II WT	skin	not detected
6		liver	not detected
7		lymph node	not detected
8		tongue	not detected
9	III MUT	skin	HPV16
10		liver	HPV16
11		lymph node	HPV16
12		tongue	HPV16
13	IV MUT	skin	HPV16
14		liver	HPV16
15		lymph node	HPV16
16		tongue	HPV16
17		oral tumour	HPV16

Legend: animal group WT wild-type FVB; MUT FVB-K14HPV16.
