# Supplementary material for: Characterization of the Human Papillomavirus 16 Oncogenes in K14HPV16 Mice: Sublineage A1 Drives Multi-Organ Carcinogenesis
Source: Int J Mol Sci. 2022 Oct 15;23(20):12371. doi: 10.3390/ijms232012371 (PMC9604181; doi:10.3390/ijms232012371)
Supplement: Supplementary file 1 [file ijms-23-12371-s001.zip › Supplementary Table S2. Genome sequences of representative strains from HPV16.pdf]

Supplementary Table S2. Genome sequences of representative strains from HPV16 lineages and sublineages include in the analysis.

GenBank	Lineage	Sublineage	Name
K02718	A	A1	European (E)
AF536179	A	A2	European (E)
HQ644236	A	A3	European (E)
AF534061	A	A4	Asian, E(As)
AF536180	B	B1	African-1, Afr1a
HQ644298	B	B2	African-1, Afr1b
AF472509	C	-	African-2, Afr2a
HQ644257	D	D1	North American (NA)1
AY686579	D	D2	Asian–American (AA)2
AF402678	D	D3	Asian–American (AA)1
